# Endoscopic evaluation technique using a camera port for valve-sparing root reconstruction

**DOI:** 10.1093/icvts/ivad039

**Published:** 2023-02-14

**Authors:** Yusuke Kawasaki, Etsuro Suenaga, Taro Nakatsu, Kenji Minatoya

**Affiliations:** Department of Cardiovascular Surgery, Kansai Electric Power Hospital, Osaka, Japan; Department of Cardiovascular Surgery, Kansai Electric Power Hospital, Osaka, Japan; Department of Cardiovascular Surgery, Kansai Electric Power Hospital, Osaka, Japan; Department of Cardiovascular Surgery, Graduate School of Medicine, Kyoto University, Kyoto, Japan

**Keywords:** Valve-sparing root reconstruction, Balloon blunt-tip system, Aortic valve evaluation

## Abstract

Intraoperative aortic valve evaluation should be accurate in valve-sparing root replacement to minimize postoperative aortic valve regurgitation. Ascending aorta de-clamping and weaning of cardiopulmonary bypass are required in intraoperative transoesophageal echocardiography. Aortic valve endoscopy aids in the magnification of structures and enables image sharing within the operative team. While a rigid endoscope and saline infusion line are directly inserted from the Valsalva graft end, a Kelly clamp is needed for graft gap closure, affecting the valve morphology due to graft deformation. The accurate inner pressure of the neo-Valsalva sinus cannot be measured in this method. We propose a technique to accurately evaluate aortic valve conformation using a balloon blunt-tip system that enables aortic valve evaluation under the measured pressure and without Valsalva graft deformation.

## INTRODUCTION

Valve-sparing root replacement is advantageous over the Bentall technique and provides excellent long-term results [1]. Intraoperative valve evaluation should be accurate to minimize postoperative aortic regurgitation (AR). Valvular prolapse or elongation can be overestimated during macroscopic evaluation because the valve and neo-Valsalva sinus (NVS) are unloaded and released by perfusion pressure [2].

**Figure 1:**
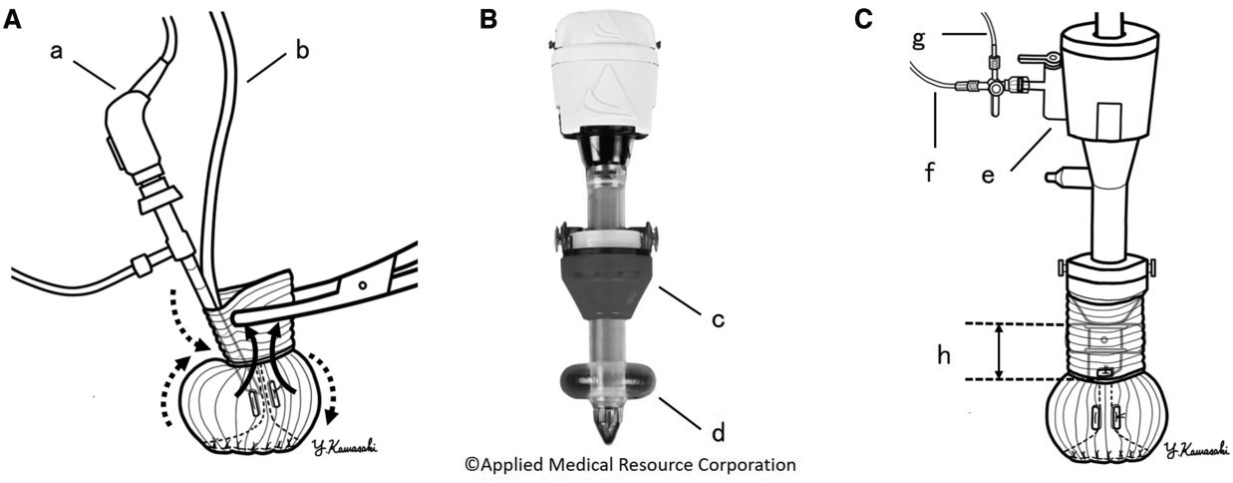
(**A**) Conventional endoscopic valvular evaluation. Neo-Valsalva sinus is deformed by clamping (straight arrow) and endoscope operation (wavy arrow): (a) endoscope and (b) saline infusion line. (**B**) Balloon blunt-tip system: (c) softgel cone and (d) balloon. (**C**) Endoscopic evaluation using the balloon blunt-tip system with a stopcock (e) connected with saline infusion (f) and manometric lines (g). The balloon and top of the port are 2 cm apart (h).

In conventional endoscopic evaluation, the endoscope and saline infusion line are directly inserted from the Valsalva graft end, and Kelly clamps are used for gap closure. However, observing the natural aortic valve morphology is difficult because NVS is deformed by clamping and endoscope operation (Fig. 1A). Furthermore, the inner pressure cannot be measured accurately.

The Kii balloon blunt-tip (BBT) system (Applied Medical Resource, Rancho Santa Margarita, CA, USA) is a camera port used for laparoscopic surgery (Fig. 1B). With a balloon at the tip and a movable softgel cone, it is used for NVS closure with the endoscope placed at the centre of the graft (Fig. 1C). This system has a stopcock that enables objective root pressure monitoring. Herein, we report a case of BBT-aided endoscopic evaluation.

## CASE PRESENTATION

A 60-year-old man, diagnosed with non-communicating DeBakey type-II aortic dissection, was referred to our hospital. A dilated aortic root and ascending aorta with tears were observed on four-dimensional computed tomography angiography. Echocardiography revealed mild-to-moderate AR. After performing median sternotomy and establishing cardiopulmonary bypass, an aortic cross-clamp was placed near the innominate trunk to induce cardiac arrest. The aortic annulus diameter and non-coronary cusp-left coronary cusp commissure height were 26 and 24 mm, respectively. A 26-mm Gelweave™ Valsalva graft (Terumo Aortic, Tokyo, Japan) was selected.

After partial aortic arch replacement, the root reconstruction was resumed. After placing the Valsalva graft, we evaluated the aortic valve using the BBT system. The NVS was marked to orient the valvular cusps. The balloon and softgel cone were positioned to facilitate closure; the inner BBT cylinder was removed before insertion into the graft. The balloon was gently inflated to fix the BBT on the graft tubing and to close the NVS; a rigid endoscope was inserted.

**Figure 2:**
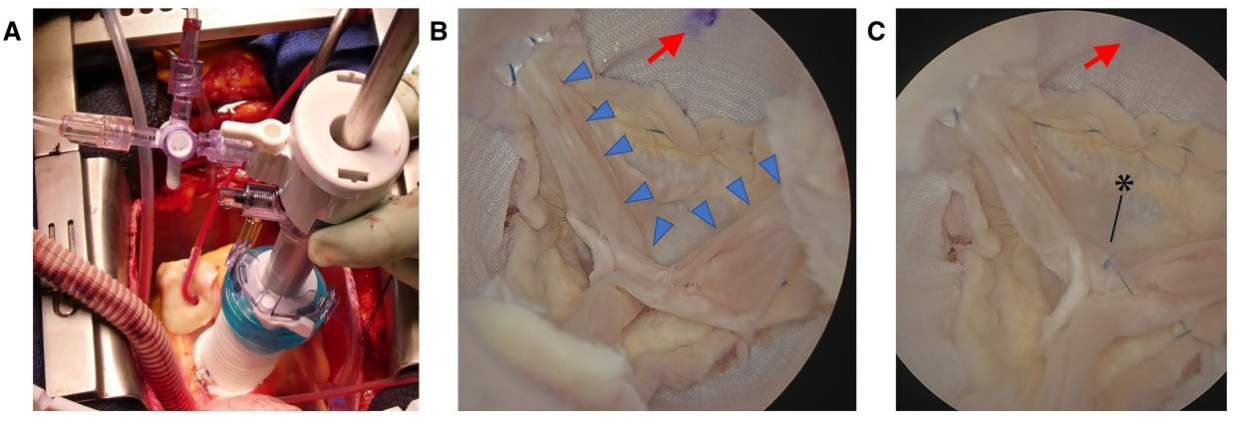
(**A**) Intraoperative image of endoscopic evaluation with the balloon blunt-tip system. (**B** and **C**) Endoscopic view of the aortic valve shows the neo-Valsalva sinus attached to the remnant right coronary cusp (arrow). (B) Minor prolapses at right coronary cusp (arrowheads) and (C) central plication (asterisk) applied on right coronary cusp.

Saline infusion and manometric lines were connected to stopcock of the BBT system. Saline was infused into the NVS to achieve a 70-mmHg pressure (Fig. 2A). The aortic valve was endoscopically evaluated. Minor prolapse at the right coronary cusp was observed (Fig. 2B); valvular coaptation was improved by central plication (Fig. 2C) video 1.

Intraoperative transoesophageal and postoperative transthoracic echocardiography revealed no residual AR.

## DISCUSSION

The BBT system is universally used in laparoscopic surgery. Balloon size can be regulated according to the graft diameter and the graft can be sufficiently closed. Using the balloon and softgel cone together, the distal end is closed without deformation. The system’s stopcock facilitates saline infusion into NVS and simultaneously measures inner pressure. This technique allows us to evaluate the morphology of aortic valve not only without graft deforming but also under artificial diastolic pressure. That provided the opportunity to correct cusp prolapse or commissure fixed position and might improve the quality and increase the reproducibility of valve repair.

To avoid mechanical injury to the aortic valve, the graft’s tube portion should be maintained above the commissure fixation point (Fig. 1C) and 25 mm long when fully extended. As this portion shrinks, it is not sufficiently long to suture the second row or evaluate the re-implanted valve. Although the tube portion can be left a little longer, a running suture in the second row makes valvular fixation easier.

## CONCLUSION

The BBT system facilitates simple and accurate physiological evaluation of valve-sparing root replacement.

## Data avaliabillity

Data sharing is not applicable to this article as no datasets were generated or analyzed during the current study.
